# Circulating tumor DNA landscape and prognostic impact of acquired resistance to targeted therapies in cancer patients: a national center for precision medicine (PRISM) study

**DOI:** 10.1186/s12943-023-01878-9

**Published:** 2023-11-04

**Authors:** Arnaud Bayle, Laila Belcaid, Lola-Jade Palmieri, Diego Teysonneau, Sophie Cousin, Mariella Spalato-Ceruso, Mihaela Aldea, Damien Vasseur, Melissa Alame, Laura Blouin, Isabelle Soubeyran, Claudio Nicotra, Maud Ngocamus, Antoine Hollebecque, Yohann Loriot, Benjamin Besse, Ludovic Lacroix, Etienne Rouleau, Fabrice Barlesi, Fabrice Andre, Antoine Italiano

**Affiliations:** 1 DITEP, Gustave Roussy, Villejuif, France; 2 Department of Oncology, Copenhagen University Hospital, Rigshospitalet, Denmark; 3 Department of Medicine, Institut Bergonié, Bordeaux, France; 4 Department of Medicine, Gustave Roussy, Villejuif, France; 5 Department of Biopathology, Gustave Roussy, Villejuif, France; 6 Department of Biopathology, Institut Bergonié, Bordeaux, France; 7 Faculty of Medicine, Paris Saclay University, Kremlin-Bicêtre, France; 8 Faculty of Medicine, University of Bordeaux, Bordeaux, France

**Keywords:** ctDNA, Targeted therapy, Resistance biomarkers, Next-generation sequencing

## Abstract

**Background:**

Despite the effectiveness of the various targeted therapies currently approved for solid tumors, acquired resistance remains a persistent problem that limits the ultimate effectiveness of these treatments. Polyclonal resistance to targeted therapy has been described in multiple solid tumors through high-throughput analysis of multiple tumor tissue samples from a single patient. However, biopsies at the time of acquired resistance to targeted agents may not always be feasible and may not capture the genetic heterogeneity that could exist within a patient.

**Methods:**

We analyzed circulating tumor DNA (ctDNA) with a large next-generation sequencing panel to characterize the landscape of secondary resistance mechanisms in two independent prospective cohorts of patients (STING: n = 626; BIP: n = 437) with solid tumors who were treated with various types of targeted therapies: tyrosine kinase inhibitors, monoclonal antibodies and hormonal therapies.

**Results:**

Emerging alterations involved in secondary resistance were observed in the plasma of up 34% of patients regardless of the type of targeted therapy. Alterations were polyclonal in up to 14% of patients. Emerging ctDNA alterations were associated with significantly shorter overall survival for patients with some tumor types.

**Conclusion:**

This comprehensive landscape of genomic aberrations indicates that genetic alterations involved in secondary resistance to targeted therapy occur frequently and suggests that the detection of such alterations before disease progression may guide personalized treatment and improve patient outcome.

**Supplementary Information:**

The online version contains supplementary material available at 10.1186/s12943-023-01878-9.

## To the editor

The development of targeted therapies has revolutionized the systemic approach to the treatment of cancer. Indubitably, hormone receptor targeting is one of the earliest examples of targeted therapy and has significantly affected the survival of patients with breast and prostate cancer, which are ‘lineage-addicted’ to estrogen receptor and androgen receptor, respectively. Therapies targeting mutated or overexpressed kinases were developed more recently and showed improvement in patient outcome in a variety of tumor types, including non-small cell lung cancer (NSCLC), melanoma, colorectal cancer, and gastrointestinal stromal tumors.

Responses to these generally well-tolerated therapies often last several months and sometimes several years. However, almost all patients treated with targeted therapies eventually demonstrate progression of their disease, a clinical setting described as acquired resistance.

Secondary alterations of the oncogene drug target and mutation of alternate components of oncogene-induced signaling pathways have been described as crucial mechanisms of acquired resistance to targeted therapies. However, their true incidence in the real-life setting and their impact on patient outcome are still unknown. Indeed, although genetic analysis of tumor tissue is the standard technique for identifying DNA alterations in malignancies, this approach has several shortcomings, including invasiveness and an inability to capture intratumor spatial and temporal heterogeneity. In contrast, next-generation sequencing (NGS) of circulating tumor DNA (ctDNA) is a method that is increasingly being used for genomic profiling of cancer and has several advantages in comparison with NGS of tissue biopsies: it is a noninvasive method, is easily achievable and repeatable, and may provide a more holistic view of actionable mutation targets in a patient’s disease, particularly in the case of treatment resistance 1.

## Methods

### Study design and procedure

Patients were included in two independent ongoing precision medicine studies (STING, NCT04932525, sponsor: Gustave Roussy; BIP, NCT02534649, sponsor: Institut Bergonié). Patients were eligible if they (i) presented a locally advanced, unresectable, or metastatic solid tumor and (ii) were being treated with targeted therapy as per standard of care at the time of plasma sampling for ctDNA profiling. All patients provided written consent for the genomic analyses. This study was approved by the Institutional Review Board.

### Genomic analysis

Genomic analysis was performed for each patient by using the Foundation One Liquid CDx Assay (324 genes, tumor mutational burden [TMB], microsatellite instability) 2. The FoundationOne Liquid CDx (F1LCDx) assay is performed on circulating cfDNA isolated from plasma derived from anti-coagulated peripheral whole blood from patients with solid malignant neoplasms. Extracted cfDNA was subjected to whole-genome shotgun library construction and hybridization-based capture of 324 cancer-related genes. In this process, all coding exons of 309 genes are targeted; select intronic or noncoding regions are targeted for fifteen of these genes. Hybrid capture-selected libraries were sequenced with deep coverage using the NovaSeq® 6000 platform. Sequence data were processed using a custom analysis pipeline designed to detect genomic alterations. Only previously reported and functionally validated resistance-related alterations were considered resistance mechanisms (see supplementary Methods).

### Statistical analysis

Descriptive statistics were used to describe population characteristics. Qualitative data are expressed as numbers and percentages. Quantitative data are expressed as the mean and standard deviation or median, 1st and 3rd quartiles and range when applicable. Data were analyzed using the software R version 4.0.3.

## Results

### Characteristics and alterations of resistance in the overall STING cohort

Here, we performed ctDNA molecular profiling to assess the incidence of genomic aberrations involved in acquired resistance and their impact on the outcome of patients treated with targeted therapies in the advanced setting. To identify candidate mechanisms of acquired resistance, in each case, cell-free DNA (cfDNA) sequencing data were compared to sequencing data from pre-treatment tumor tissue to identify emergent alterations 3 (see Supplementary Methods).

**Fig. 1:**
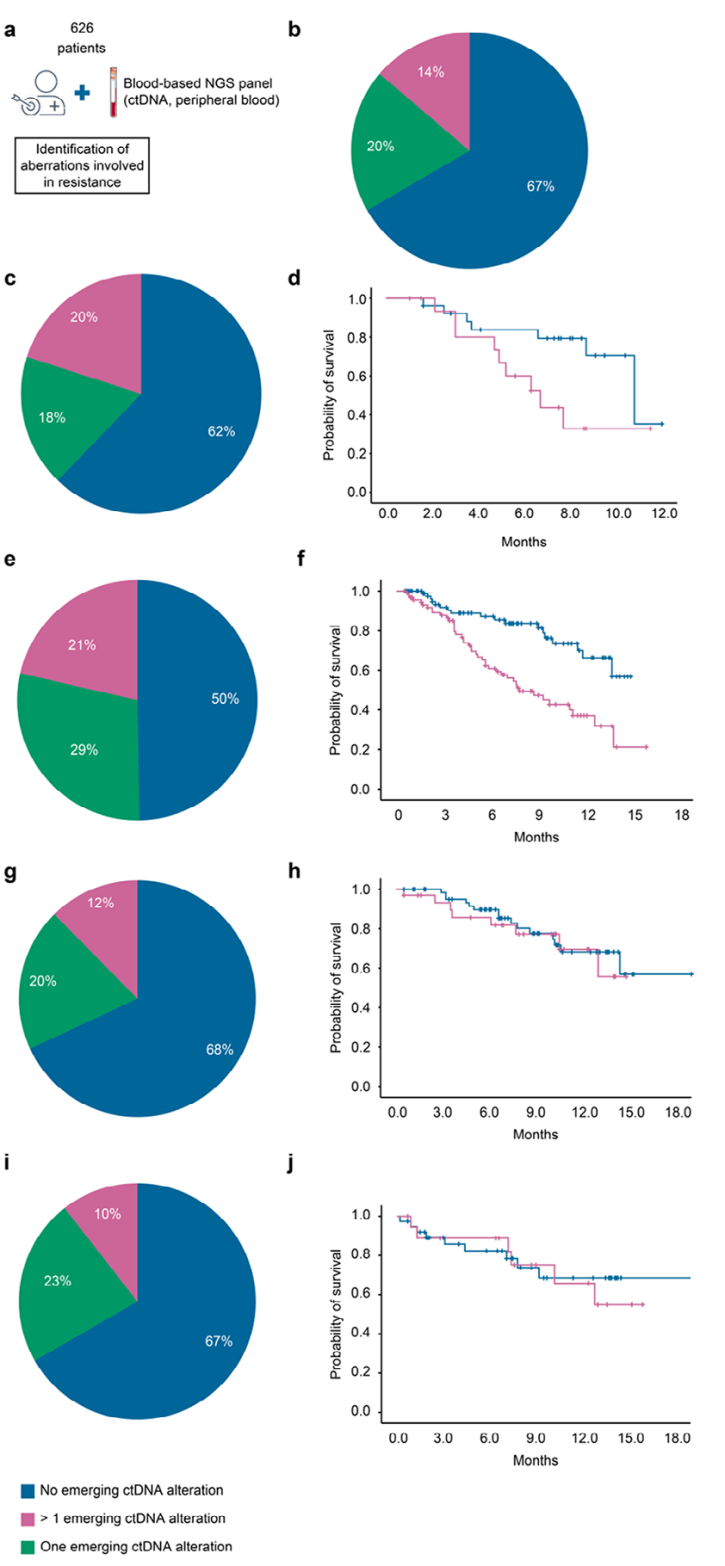
**Incidence of ctDNA emerging alterations in patients with advanced cancer and treated with targeted therapy** (**A**) 626 patients enrolled in the STING study and treated with a targeted therapy had ctDNA profiling. (**B**) Incidence of ctDNA emerging alterations in the whole cohort. (**C**) Incidence of ctDNA emerging alterations in patients with advanced colorectal cancer and receiving an anti-EGFR monoclonal antibody. (**D**) Kaplan-Meier curve of overall survival in colorectal cancer patients according to presence (red curve) or not (blue line) of at least one ctDNA emerging alteration. (**E**) Incidence of ctDNA emerging alterations in patients with advanced prostate cancer and receiving an anti-androgen hormonal therapy. (**F**) Kaplan-Meier curve of overall survival in prostate cancer patients according to presence or not of at least one ctDNA emerging alteration. (**G**) Incidence of ctDNA emerging alterations in patients with advanced breast cancer and receiving an anti-oestrogen hormonal therapy. (**H**) Kaplan-Meier curve of overall survival in breast cancer patients according to presence or not of at least one ctDNA emerging alteration (**I**) Incidence of ctDNA emerging alterations in patients with advanced non-small cell lung cancer and receiving an EGFR tyrosine kinase inhibitor. (**J**) Kaplan-Meier curve of overall survival in non-small cell lung cancer patients according to presence or not of at least one ctDNA emerging alteration

Therefore, we evaluated a prospective cohort of 626 patients with advanced cancer who were treated with a targeted therapy and enrolled in an institutional molecular profiling program (STING, NCT04932525, sponsor: Gustave Roussy, Villejuif, France) to obtain a molecular profile based on ctDNA profiling (Fig. 1A). Their characteristics are described in Table 1. ctDNA profiling identified at least one previously validated resistance alteration in 193 patients out of 626 (31%). Notably, 86 patients (14%) exhibited > 1 detectable resistance alteration (range 2–16, median 3 per patient), suggesting frequent and profound tumor heterogeneity associated with acquired resistance (Fig. 1B and Supplementary Table 1). Patients with at least 1 emerging ctDNA alteration detected had significantly lower median overall survival (OS) than patients with no mechanism of resistance: 16.2 months (95% CI 14.8–18.3) vs. 10.2 months (6.5–13.9) (p < 0.001).

**Table 1:** Patients characteristics

**STING study**		
**Number of patients**		N = 626
**Sex**	Male Female	336 53.7% 290 46.3%
**Age**	Median (range)	Med = 64 (19; 90)
**Cancer type**	Colorectal cancer Non-small cell lung cancer Thyroid cancer Breast cancer Head and neck cancer Prostate cancer Others	58 9.3% 10316.4% 23 3.7% 12619.8% 304.8% 18830.0% 9816%
**Type of treatment**	Tyrosine kinase inhibitor Monoclonal antibody Hormonal therapy	17628.1% 13822.0% 31249.9%
**PS (at ctDNA)**	0 1 2 Missing values	22636.1% 28946.2% 457.2% 6610.5%
**BIP study**		
**Number of patients**		N = 437
**Sex**	Male Female	155 35.5% 282 64.5%
**Age**	Median (range)	Med = 66 (21 ; 87)
**Cancer type**	Colorectal cancer Non-small cell lung cancer Breast cancer Prostate cancer	62 14.2% 14 3.2% 25157.4% 1102.5%
**Type of treatment**	Tyrosine kinase inhibitor Monoclonal antibody Hormonal therapy	143.2% 6214.2% 36182.6%
**ECOG Performance Status (at ctDNA assesment)**	0 1 2	17,039% 21,850% 4911%

### Emerging alterations in colorectal cancer patients: insights from subgroup analysis in the STING and BIP studies

We then analyzed the patterns and prognostic impact of ctDNA alterations in 4 specific subgroups of patients enrolled in the STING study: *KRAS*/*BRAF* wild-type (WT) colorectal cancer patients treated with anti-EGFR monoclonal antibodies, prostate cancer patients treated with antiandrogen therapy, hormone receptor-positive breast cancer patients treated with an aromatase inhibitor-based regimen and EGFR-mutated NSCLC patients treated with an anti-EGFR tyrosine kinase inhibitor. To confirm the findings observed in the STING study, we analyzed the same subgroups of patients enrolled in an independent precision medicine study (BIP, NCT02534649, sponsor: Institut Bergonié, Bordeaux, France). Their characteristics are described in Table 1.

**Fig. 2:**
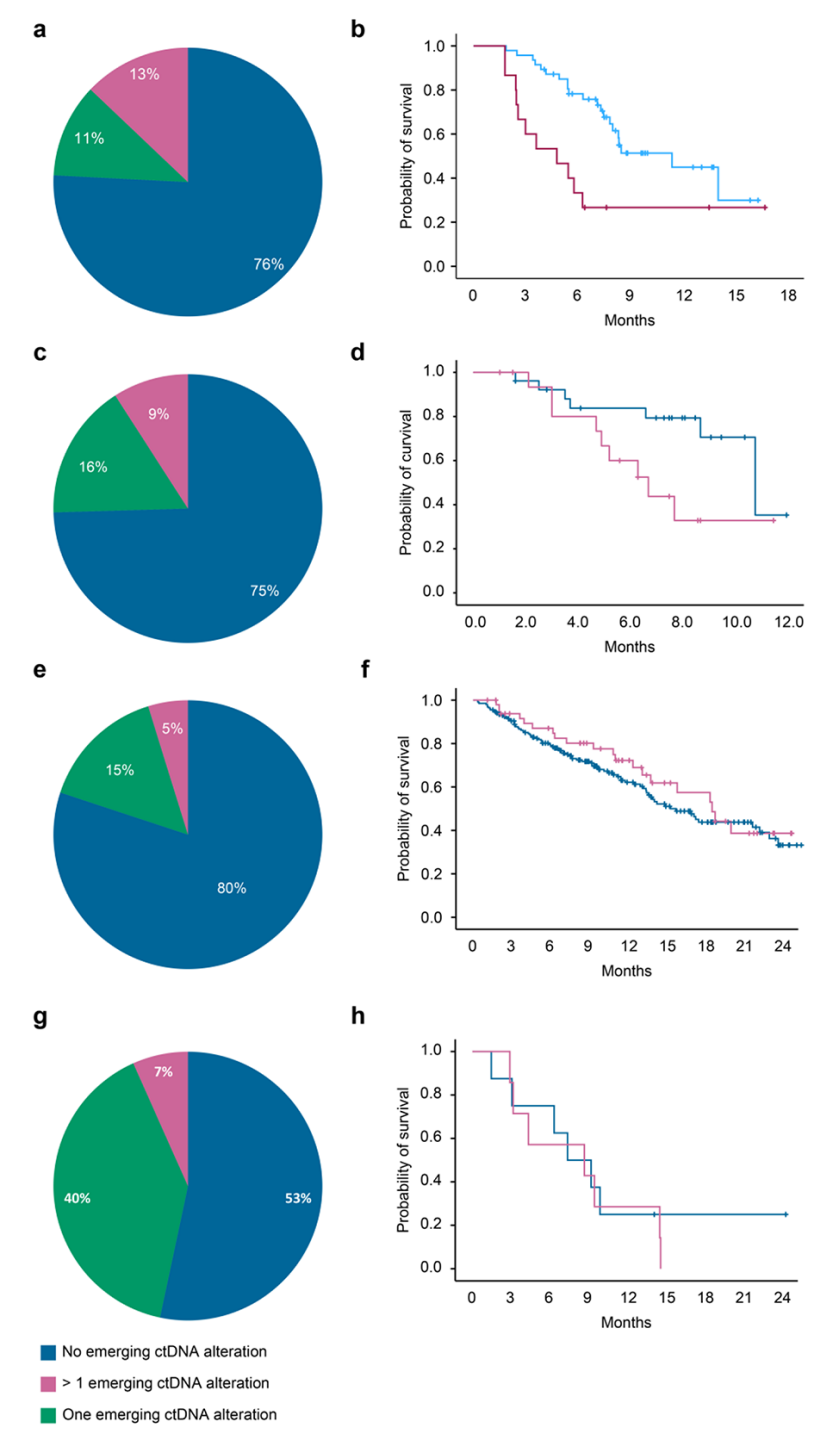
**Incidence of ctDNA emerging alterations in patients with advanced cancer and treated with targeted therapy in the BIP study.** (**A**) Incidence of ctDNA emerging alterations in patients with advanced colorectal cancer and receiving an anti-EGFR monoclonal antibody. (**B**) Kaplan-Meier curve of overall survival in colorectal cancer patients according to presence or not of at least one ctDNA emerging alteration. (**C**) Incidence of ctDNA emerging alterations in patients with advanced prostate cancer and receiving an anti-androgen hormonal therapy. (**D**) Kaplan-Meier curve of overall survival in prostate cancer patients according to presence or not of at least one ctDNA emerging alteration. (**E**) Incidence of ctDNA emerging alterations in patients with advanced breast cancer and receiving an anti-oestrogen hormonal therapy. (**F**) Kaplan-Meier curve of overall survival in breast cancer patients according to presence or not of at least one ctDNA emerging alteration (**G**) Incidence of ctDNA emerging alterations in patients with advanced non-small cell lung cancer and receiving an EGFR tyrosine kinase inhibitor. (**H**) Kaplan-Meier curve of overall survival in breast cancer patients according to presence or not of at least one ctDNA emerging alteration

In the STING study, ctDNA sequencing allowed the identification of genetic alterations involved in acquired resistance to EGFR-specific antibodies in 17 patients (38%) out of 45 with colorectal cancer (Fig. 1C and Supplementary Table 1). The most frequent emergent aberrations observed in our cohort of patients were RAS pathway mutations in 10 patients (22%). Among them, 5 patients (11%) harbored ≥ 2 variants (up to eight), and 2 also had KRAS amplification (13.3%). Other emergent alterations associated with resistance to anti-EGFR treatment observed in our cohort included *EGFR* mutations (n = 6, with 3 patients harboring ≥ 2 variants), *MET* amplification/mutation (n = 4), *PI3KCA* mutation (n = 3), *HER3* mutation (n = 1), and *BRAF* fusion (n = 1). Overall, 9 patients (20%) exhibited polyclonal resistance. The median OS time was significantly lower in patients with at least one identified emerging ctDNA alteration: 6.7 (95% CI 4.2–9.1) vs. 10.8 months (95% CI 7.8–13.8), p = 0.04 (Fig. 1D). Similar patterns were observed in the BIP cohort, with 15 patients (24%) out of 62 harboring genetic aberrations involved in resistance; *KRAS* and *EGFR* gene alterations were the two most frequent. Resistance was polyclonal in 8 patients (12.9%) (Fig. 2A and Supplementary Table 2). The median OS time was like that observed in the STING study: 4.8 (95% CI 1.7–7.9) vs. 11.4 months (95% CI 5.8–17) in patients with no mechanism of resistance or at least one identified mechanism of resistance, respectively, p = 0.004 (Fig. 2B).

### Emerging alterations in prostate cancer patients: insights from subgroup analysis in the STING and BIP studies

At least one emerging ctDNA alteration involved in resistance to anti-androgen therapy was identified in 94 patients with prostate cancer (50%) out of 188 included in the STING study. *AR* amplifications were identified in 57 patients (60.6% of AR-altered patients and 30.3% overall) included in the STING cohort (Fig. 1E and Supplementary Table 1). A mutation or rearrangement in *AR* was identified in 59 patients (31.4%). Among them, 28 patients (14.9%) harbored ≥ 2 variants (up to five), and 25 harbored an *AR* amplification (13.3%). Hotspots are essentially located in the ligand binding domain and are involved in resistance to androgen receptor signaling inhibitors. The most frequent mutations included W742C/F (bicalutamide resistance) (n = 4), H875Y (n = 9), F877L (n = 3) and T878A (n = 25) (bicalutamide/enzalutamide/apalutamide resistance and promiscuous activation by progesterone), and L702H (resistance to abiraterone/enzalutamide, as well as the AR proteolysis-targeting chimera ARV-110, and activation by corticosteroids) (n = 25). Less common *AR* resistance mutations were V716M (n = 3), G689A (n = 1) and D891H (n = 2); such mutations have been previously described in ctDNA from patients with metastatic castration-resistant prostate cancer (mCRPC) who progressed on bicalutamide or abiraterone. Additional AR gene rearrangements that consisted of deletions or inversions impacting the genomic segment containing AR exons 5–7 and have already been reported in patient-derived xenograft models were identified in 5 patients. Overall, polyclonal resistance was observed in 40 patients (21%). The median OS time was significantly lower in patients with at least one identified emerging ctDNA alteration than in those with no identified alterations: 7.9 months vs. not reached (95% CI 5.5–10.3), p < 0.001 (Fig. 1F). In the BIP study, at least one emerging ctDNA alteration involved in resistance to anti-androgen therapy was identified in 28 patients (25%) out of 112. (Fig. 2C and Supplementary Table 2). AR amplifications were identified in 27 patients (61.4% of AR-altered patients and 24.5% overall). As observed in the STING study, the most frequent mutation observed was AR T878A. Among patients with mutation or rearrangement in *AR*, 5 patients (4.5%) harbored ≥ 2 variants (up to five) and 2 harbored an AR amplification (1.9%). Polyclonal mechanisms of resistance were identified in 10 patients (9%). Median OS was not reached in patients with no emerging mutation or patients with at least one emerging ctDNA alteration, but the survival time was significantly lower in patients with at least one emerging ctDNA alteration (p < 0.001) (Fig. 2D).

### Emerging alterations in breastl cancer patients: insights from subgroup analysis in the STING and BIP studies

*ESR1* mutations, which represent a key driver of endocrine therapy resistance, were identified in 31 patients (32%) out of 97 with advanced breast cancer who were treated with aromatase inhibitors in the STING study (Fig. 1G Supplementary Table 1). Among them, 12 patients (12%) harbored ≥ 2 variants (up to three). In the BIP study, ESR1 mutations were identified in 50 patients (20%) out of 250 (Fig. 2E and Supplementary Table 2). Eight patients (3.2%) harbored ≥ 2 variants (up to 7). In the STING and BIP studies, no survival difference was observed between patients harboring ctDNA emerging alterations and those who did not harbor such alterations (Fig. 1H F).

### Emerging alterations in NSCLC patients: insights from subgroup analysis in the STING and BIP studies

In the STING study, ctDNA sequencing allowed the identification of genetic alterations involved in acquired resistance to anti-EGFR tyrosine kinase inhibitors in 19 patients (33.3%) out of 57 with EGFR-addicted NSCLC (Fig. 1I and Supplementary Table 1). The most frequent aberrations observed in our cohort of patients were the emergence of EGFR pathway mutations in 9 patients (15.8%). Other emergent alterations associated with resistance to anti-EGFR treatment observed in our cohort included *PI3KCA* mutation, HER3 amplification and fusions involving other key oncogene drivers, such as RET (n = 1), BRAF (n = 1) and NTRK1 (n = 1). Overall, the mechanisms of resistance were polyclonal in 8 patients (14%). In the BIP study, emerging alterations involved in resistance were observed in 4 patients (25%) out of 16 and included EGFR mutations, PI3KCA mutation and AKT amplifications (Fig. 2G and Supplementary Table 2). In both the STING and BIP studies, no survival difference was observed between patients harboring ctDNA emerging alterations and those who did not harbor such mutations (Fig. 1J H).

## Discussion

We report here a comprehensive study investigating the incidence of genetic aberrations involved in resistance to targeted therapies detected through ctDNA sequencing of patients with advanced cancer. By analyzing two independent cohorts of patients, we were able to demonstrate that such aberrations are present prior to disease progression and are polyclonal in up to 14% of patients. This polyclonality illustrates the utility of ctDNA sequencing compared to tumor tissue sequencing in characterizing the tumor molecular profile. The clinical impact of the detection of genetic aberrations involved in resistance through ctDNA profiling remains to be demonstrated through randomized clinical trials. Our study suggests that further exploration into using ctDNA sequencing results to potentially guide therapeutic decisions might be of value. In colorectal cancer, the results of the CRICKET study presented the first evidence that RAS WT status, as assessed by liquid biopsy, could represent an appropriate tool to select patients who might benefit from anti-EGFR rechallenge therapies 4. In this proof-of-concept study, the investigators evaluated the safety and efficacy of a rechallenge with 3rd line anti-anti-EGFR treatment in patients who presented secondary resistance after initial benefit from a 1st line anti-EGFR/irinotecan-based regimen and 2nd line bevacizumab and oxaliplatin. They demonstrated that both progression-free survival (PFS) and OS were improved in patients with baseline *KRAS* WT ctDNA compared to baseline *KRAS* mutant ctDNA (4.0 vs. 1.9 months; hazard ratio, HR, 0.44; 95% CI, 0.18–098; p = 0.03; 12.5 vs. 5.2 months; HR, 0.58; 95% CI, 0.22–1.52; p = 0.24). In our study, which included patients with *KRAS* WT colorectal cancer receiving anti-EGFR monoclonal antibodies, the detection of emerging resistant ctDNA aberrations was associated with significantly worse OS in two independent cohorts. This suggests that switching from an anti-EGFR-based regimen to another line of treatment upon early identification of the resistance mutation in plasma—before disease progression—may improve patient outcome and deserves further investigation in a randomized setting.

Androgen deprivation therapy has been a cornerstone of treatment for patients with prostate cancer since early 1940. Several new potent androgen axis inhibitors such as enzalutamide, apalutamide and darolutamide abiraterone acetate are now being approved for castration-sensitive metastatic disease 5. The novel antiandrogens work as classic androgen receptor antagonists with high potency, and abiraterone is a CYP17 inhibitor that decreases androgen synthesis. After being approved for the treatment of mCRPC, novel androgen axis inhibitors (e.g., enzalutamide, apalutamide, darolutamide) are now being developed for metastatic castration‐sensitive prostate cancer 6. Several of the alterations we have identified in our study have a direct impact on the efficacy of these drugs. For instance, the F877L mutation confers resistance to both enzalutamide and apalutamide by converting them into agonists 7. A similar phenomenon has been described with the W742C/L, T878S, S889G, and D891H mutations, which can result in resistance to bicalutamide by converting this drug to an agonist 7. The significantly shorter survival times observed in patients harboring *AR* alterations also advocates for the implementation of serial ctDNA sampling to identify mutational mechanisms of drug resistance to antiandrogen therapy. Indeed, this can result in timelier and more appropriate therapeutic decisions that can improve patient outcomes. For instance, responses to withdrawal of enzalutamide, bicalutamide, nilutamide, and flutamide are well described 8–11, but the underlying molecular defects related to these observations are unknown. Our results showing a significant impact of the emergence of ctDNA resistance aberrations on OS support the application of precision medicine and encourage randomized studies aiming to demonstrate that tailoring therapy based on ctDNA monitoring in patients with advanced prostate cancer can improve patient outcome.

A significant proportion of patients with advanced breast cancer and treated with aromatase inhibitors displayed *ESR1* mutations that have been associated with resistance to aromatase inhibitors 12. Our results corroborate those of previous studies demonstrating that ESR1 mutations are detectable prior to progression at a median of 3–6 months beforehand in up to 86% of breast cancer patients treated with aromatase inhibitors. Interestingly, we did not find a detrimental effect on OS associated with the detection of such mutations. One explanation may be related to the fact that most of the patients were treated with a combination of aromatase inhibitor and CDK4 inhibitor, which represents the current standard of care. Another potential explanation could be that patients with hormone receptor-positive breast cancer have a substantially prolonged OS after progression on hormone-based therapy due to the availability of several active lines of treatment. However, this does not preclude the potential utility of monitoring the emergence of *ESR1* mutations in the ctDNA of patients treated with a combination of aromatase inhibitor and CDK4 inhibitor, as illustrated by the recently reported results of the PADA-1 study 13. PADA-1 was a phase III trial in which investigators used a plasma-based assay to identify *ESR1* mutations in patients being treated with an aromatase inhibitor plus palbociclib. Patients found to have a mutation prior to disease progression were randomly assigned to continue treatment or switch to fulvestrant plus palbociclib. Interestingly, switching therapy resulted in a doubling of median PFS time, from 5.7 months with standard treatment to 11.9 months (hazard ratio = 0.61; P = 0.005). Data regarding OS are not yet available and are eagerly awaited.

We found that up to 33% of patients with EGFR-mutated NSCLC harbored newly acquired mutations involved in resistance to EGFR TKIs at the time of ctDNA sequencing, and the emergence of such mutations was not associated with OS. This lack of prognostic impact may be related to the availability of highly efficient treatment in patients with acquired mutations such as osimertinib for patients harboring the *EGFR* T790M mutation 14. Masip et al. 15 recently reported the results of the APPLE study, which is a randomized, noncomparative, open-label, 3-arm, phase II study aiming to evaluate the impact of sequential plasma EGFR T790M monitoring in patients with EGFR-mutated NSCLC. In one arm, patients were treated with first-line gefitinib (250 mg daily) until emergence of ctDNA T790M-positive status. The treatment was then switched to osimertinib (80 mg daily) until second disease progression according to RECIST; in the other arm, patients received first-line gefitinib (250 mg daily) until disease progression according to RECIST and then switched to osimertinib (80 mg daily) until second disease progression according to RECIST. As observed in our study, ctDNA sequencing allowed the identification of mechanisms of resistance in a significant proportion of cases. Indeed, 17% of patients treated with gefitinib had emerging *EGFR* T790M mutation occurring before progression as per RECIST. However, the median OS was not significantly different between the two arms (18 months OS, 87% vs. 77%), suggesting that further studies are needed to clarify the role of ctDNA monitoring in this setting.

One main limitation of our study is that ctDNA profiling cannot capture all the complexity of the processes involved in resistance to targeted therapies. For instance, this approach does not allow the detection of epigenetic alterations or functional activation of redundant kinases involved in bypass pathways. However, thanks to a robust methodology (multicentric setting with independent cohorts, large sample size, ctDNA profiling with an FDA-approved large NGS panel assay), our findings demonstrate the complex and heterogeneous molecular mechanisms of resistance in cancer patients and illustrate the great potential of ctDNA profiling for guiding treatment decision-making and improving prognosis in clinical practice.

### Electronic supplementary material

Below is the link to the electronic supplementary material.

Supplementary Material 1

Supplementary Material 2

Supplementary Material 3

## Data Availability

Individual participant data that underlie the results reported in this article will be available after deidentification beginning 24 months and ending 48 months following article publication to researchers who will provide a methodologically sound proposal. Requests should be sent to the corresponding author.

## List of abbreviations

**ctDNA**: circulating tumor DNA
**FDA**: Food and Drug Administration
**mCRPC**: metastation castration-resistant prostate cancer
**NSCLC**: non-small cell lung cancer
**OS**: overall survival
**PFS**: progression-free survival
**TKIs**: tyrosine kinase inhibitors
**WT**: wild-type
