# Supplementary material for: Circulating tumor DNA landscape and prognostic impact of acquired resistance to targeted therapies in cancer patients: a national center for precision medicine (PRISM) study
Source: Mol Cancer. 2023 Nov 4;22:176. doi: 10.1186/s12943-023-01878-9 (PMC10625178; doi:10.1186/s12943-023-01878-9)
Supplement: Supplementary file 1 — Supplementary Material 1 [file 12943_2023_1878_MOESM1_ESM.docx]

**SUPPLEMENTARY METHODS**

***Characterization of acquired mutations***

To identify candidate mechanisms of acquired resistance, in each case, cell-free DNA (cfDNA) sequencing data were compared to sequencing data from pre-treatment tumor tissue to identify emergent alterations ^3^. Only previously reported and functionally validated resistance-related alterations were considered resistance mechanisms^1-9^.

***Supplementary references***

1. Misale, S., Di Nicolantonio, F., Sartore-Bianchi, A., Siena, S. & Bardelli, A. Resistance to Anti-EGFR therapy in colorectal cancer: from heterogeneity to convergent evolution. *Cancer Discov.* 4, 1269–1280 (2014).

2. Misale, S. et al. Emergence of KRAS mutations and acquired resistance to anti-EGFR therapy in colorectal cancer. *Nature* 486, 532–536 (2012).

3. Morgillo, F., Della Corte, C. M., Fasano, M. & Ciardiello, F. Mechanisms of resistance to EGFR-targeted drugs: lung cancer. *ESMO Open* 1, e000060 (2016).

4. Ricordel, C., Friboulet, L., Facchinetti, F. & Soria, J. C. Molecular mechanisms of acquired resistance to third-generation EGFR-TKIs in EGFR T790M-mutant lung cancer. *Ann. Oncol.* 29, i28–i37 (2018).

5. Robinson, D. et al. Integrative clinical genomics of advanced prostate cancer. *Cell* 161, 1215–1228 (2015).

6. Quigley, D. A. et al. Genomic hallmarks and structural variation in metastatic prostate cancer. *Cell* 174, 758–769.e759 (2018).

7. Joseph, J. D. et al. A clinically relevant androgen receptor mutation confers resistance to second-generation antiandrogens enzalutamide and ARN-509. *Cancer Discov.* 3, 1020–1029 (2013).

8. Korpal, M. et al. An F876L mutation in androgen receptor confers genetic and phenotypic resistance to MDV3100 (Enzalutamide). *Cancer Discov.* 3, 1030–1043 (2013).

9. Herzog, S. K. & Fuqua, S. A. W. ESR1 mutations and therapeutic resistance in metastatic breast cancer: progress and remaining challenges. *Br. J. Cancer* 126, 174–186 (2021).

10. Lièvre, A. et al. *KRAS* mutation status is predictive of response to cetuximab therapy in colorectal cancer. *Cancer Res.* 66, 3992–3995 (2006).

11. Chuang, J. et al. MAP2K1 mutations in advanced colorectal cancer predict poor response to anti-EGFR therapy and to vertical targeting of MAPK pathway. *Clin. Colorectal Cancer* 20, 72–78 (2021).

12. Wang, H., Liang, L., Fang, J. Y. & Xu, J. Somatic gene copy number alterations in colorectal cancer: new quest for cancer drivers and biomarkers. *Oncogene* 35, 2011–2019 (2015).

13. Favazza, L. A. et al. KRAS amplification in metastatic colon cancer is associated with a history of inflammatory bowel disease and may confer resistance to anti-EGFR therapy. *Mod. Pathol.* 33, 1832–1843 (2020).

14. Montagut, C. et al. Identification of a mutation in the extracellular domain of the epidermal growth factor receptor conferring cetuximab resistance in colorectal cancer. *Nat. Med.* 18, 221–223 (2012).

15. Bardelli, A. et al. Amplification of the MET receptor drives resistance to anti-EGFR therapies in colorectal cancer. *Cancer Discov.* 3, 658–673 (2013).

16. Xu, J.-M. et al. *PIK3CA* mutations contribute to acquired cetuximab resistance in patients with metastatic colorectal cancer. *Clin. Cancer Res.* 23, 4602–4616 (2017).

17. Zhang, L. et al. ERBB3/HER2 signaling promotes resistance to EGFR blockade in head and neck and colorectal cancer models. *Mol. Cancer Ther.* 13, 1345–1355 (2014).

18. Stangl, C. et al. Diverse *BRAF* gene fusions confer resistance to EGFR-targeted therapy via differential modulation of BRAF activity. *Mol. Cancer Res.* 18, 537–548 (2020).

19 Lallous, N. et al. Functional analysis of androgen receptor mutations that confer anti-androgen resistance identified in circulating cell-free DNA from prostate cancer patients. *Genome Biol.* 17, 10 (2016).

20. Gao, X. et al. Phase 1/2 study of ARV-110, an androgen receptor (AR) PROTAC degrader, in metastatic castration-resistant prostate cancer (mCRPC). *J. Clin. Oncol.* 40, 17 (2022).

21. Sumiyoshi, T. et al. Clinical utility of androgen receptor gene aberrations in circulating cell-free DNA as a biomarker for treatment of castration-resistant prostate cancer. *Sci. Rep.* 9, 4030 (2019).

22. Azad, A. A. et al. Androgen receptor gene aberrations in circulating cell-free DNA: biomarkers of therapeutic resistance in castration-resistant prostate cancer. *Clin. Cancer Res.* 21, 2315–2324 (2015).

23. Henzler, C. et al. Truncation and constitutive activation of the androgen receptor by diverse genomic rearrangements in prostate cancer. *Nat. Commun.* 7, 13668 (2016).

24. Wu, S.-G., Chang, Y.-L., Yu, C.-J., Yang, P.-C. & Shih, J.-Y. The role of PIK3CA mutations among lung adenocarcinoma patients with primary and acquired resistance to EGFR tyrosine kinase inhibition. *Sci. Rep.* 6, 35249 (2016).

25. Yonesaka, K. et al. Anti-HER3 monoclonal antibody patritumab sensitizes refractory non-small cell lung cancer to the epidermal growth factor receptor inhibitor erlotinib. *Oncogene* 35, 878–886 (2015).

26. Piotrowska, Z. et al. Landscape of acquired resistance to osimertinib in *EGFR*-mutant NSCLC and clinical validation of combined EGFR and RET inhibition with osimertinib and BLU-667 for acquired *RET* fusion. *Cancer Discov.* 8, 1529–1539 (2018).

27. Xia, H. et al. Evidence of NTRK1 fusion as resistance mechanism to EGFR TKI in EGFR+ NSCLC: results from a large-scale survey of NTRK1 fusions in chinese patients with lung cancer. *Clin. Lung Cancer* 21, 247–254 (2020).
